# Extraintestinal pathogenic *Escherichia coli* in poultry meat products on the Finnish retail market

**DOI:** 10.1186/1751-0147-54-64

**Published:** 2012-11-16

**Authors:** Ulrike Lyhs, Ilona Ikonen, Tarja Pohjanvirta, Kaisa Raninen, Päivikki Perko-Mäkelä, Sinikka Pelkonen

**Affiliations:** 1 Ruralia Institute, University of Helsinki, Kampusranta 9C, FI-60320, Seinäjoki, Finland; 2 Research and Laboratory Department, Veterinary Bacteriology Research Unit, Finnish Food Safety Authority Evira, Neulaniementie 4, FI-70210, Kuopio, Finland; 3 Research and Laboratory Department, Production Animal and Wildlife Health, Finnish Food Safety Authority Evira, PO Box 198, FI-60101, Seinäjoki, Finland

**Keywords:** Extraintestinal pathogenic *Escherichia coli*, ExPEC, Poultry meat, Retail market, Antibiotic resistance

## Abstract

**Background:**

Extraintestinal pathogenic *Escherichia coli* bacteria (ExPEC) exist as commensals in the human intestines and can infect extraintestinal sites and cause septicemia. The transfer of ExPEC from poultry to humans and the role of poultry meat as a source of ExPEC in human disease have been discussed previously. The aim of the present study was to provide insight into the properties of ExPEC in poultry meat products on the Finnish retail market with special attention to their prevalence, virulence and phylogenetic profiles. Furthermore, the isolates were screened for possible ESBL producers and their resistance to nalidixic acid and ciprofloxacin was tested.

**Methods:**

The presence of ExPEC in 219 marinated and non-marinated raw poultry meat products from retail shops has been analyzed. One *E. coli* strain per product was analyzed further for phylogenetic groups and possession of ten virulence genes associated with ExPEC bacteria (*kpsMT* K1*, ibeA, astA, iss, irp2, papC, iucD, tsh, vat* and *cva/cv*) using PCR methods. The *E. coli* strains were also screened phenotypically for the production of extended-spectrum β-lactamase (ESBL) and the susceptibility of 48 potential ExPEC isolates for nalidixic acid and ciprofloxacin was tested.

**Results:**

*E. coli* was isolated from 207 (94.5%) of 219 poultry meat products. The most common phylogenetic groups were D (50.7%), A (37.7%), and B2 (7.7%). Based on virulence factor gene PCR, 23.2% of the strains were classified as ExPEC. Two ExPEC strains (1%) belonged to [O1] B2 *svg*+ (specific for virulent subgroup) group, which has been implicated in multiple forms of ExPEC disease. None of the ExPEC strains was resistant to ciprofloxacin or cephalosporins. One isolate (2.1%) showed resistance to nalidixic acid.

**Conclusions:**

Potential ExPEC bacteria were found in 22% of marinated and non-marinated poultry meat products on the Finnish retail market and 0.9% were contaminated with *E. coli* [O1] B2 *svg*+ group. Marinades did not have an effect on the survival of ExPEC as strains from marinated and non-marinated meat products were equally often classified as ExPEC. Poultry meat products on the Finnish retail market may have zoonotic potential.

## Introduction

*Escherichia coli* strains can be classified into three major groups: commensal strains, intestinal pathogenic strains, and extraintestinal pathogenic *E. coli* (ExPEC) strains [1]. ExPEC strains derive predominantly from *E. coli* phylogenetic group B2, and to a lesser extent D, and were defined by Johnson et al. [2] as *E. coli* isolates containing two or more of the following virulence markers: *papA* (P fimbriae structural subunit) and/or *papC* (P fimbriae assembly), *sfa/foc* (S and F1C fimbriae subunits), *afa/dr* (Dr-antigen-binding adhesins), *kpsMT II* (group 2 capsular polysaccharide units), and *iutA* (aerobactin receptor). ExPEC exist as commensals in the human intestines and can infect extraintestinal sites, such as the urinary tract, the meninges, the peritoneal cavity or lungs, and can also cause septicemia [3,4]. The transfer of ExPEC from poultry to humans and the role of poultry meat as a source of ExPEC human disease have been discussed previously. Studies by Johnson et al. [2,5-7] and Manges et al. [3] indicated that retail meat products are frequently contaminated with *E. coli*, and that poultry and pork meat may be a potential source of ExPEC strains.

There is a wide variety of poultry products available on the Finnish retail market, including fresh and modified-atmosphere-packaged (MAP) products with or without spices. Most of these products, approximately 80%, are sold marinated with salt and water- and/or oil-based, spiced sauces [8]. To the authors’ knowledge, there is no information about the prevalence of ExPEC in marinated and non-marinated poultry meat products on the Finnish retail market.

The use of antibiotic agents in food animal production has raised concern about the transmission of antibiotic-resistant bacteria to humans via the food supply. Acquired resistance to first-line antimicrobial agents has been found to increasingly complicate the management of ExPEC infections in humans. Resistance to fluoroquinolones and third- and fourth-generation cephalosporins is increasing in many countries [9-12]. Increasing numbers of extended-spectrum β-lactamase (ESBL)-producers are seen in Finnish uro-pathogenic *E. coli* (UPECs) (M. Vaara, personal communication). ESBL producing *E. coli* strains have emerged as a potential health hazard, as they have not only been identified from human clinical samples, but also from food producing animals [10,13].

The aim of the present study was to provide insight into the properties of ExPEC in poultry meat products on the Finnish retail market in order to evaluate their zoonotic potential. Special attention was given to their prevalence and the virulence and phylogenetic profiles. Furthermore, the isolates were screened for possible ESBL producers and their antibiotic resistance to nalidixic acid and ciprofloxacin was tested.

## Materials and methods

### Product description and sampling

A total of 219 retail raw chilled poultry meat products from different batches (199 chicken samples, 16 turkey samples and four samples including both chicken and turkey meat) were randomly selected between July 2006 and September 2006 from different local retail shops in Western Finland (88 samples) and between August 2006 and April 2007 in the Helsinki area (131 samples). Of all samples, 178 products were marinated and 41 were non-marinated. All products were packed in Finland, but in 31 (14.2%) samples the meat originated abroad. The term “marinated” refers to products with an oil- and/or water-based marinade and a blend of spices. All products were packed under a modified atmosphere consisting of carbon dioxide and nitrogen in different proportions, and had a shelf life of up to 10 days. All samples were transported immediately to the laboratory and kept at 4 ± 3°C until being analyzed within 24 hours of purchase.

### Culture method and identification

Each sample was aseptically removed from the package and placed in a Stomacher bag (Seward BA6041, Worthing, UK). Equal amounts of a weighed sample and Buffered Peptone Water (BPW) (LAB46, LabM, Lancashire, UK) were mixed with a minimal amount of 300 g of meat in 300 ml of BPW (LabM). The bag was shaken manually for 3 minutes and100 μl of BPW was spread onto Chromocult® Coliform Agar (CCA) (Merck, Darmstadt, Germany) plates. All plates were incubated for 24 hours at 37°C. One presumptive *E. coli* colony isolated from each product was subcultured on Tryptic Soy Agar (TSA) agar (Merck, Darmstadt, Germany), and incubated as described above. For further identification, API 20E (bio Mérieux, Marcy l`Etoile, France) was used.

### PCR methods

#### Virulence genotyping

A total of 207 *E. coli* isolates were characterized for their virulence factor genes using the polymerase chain reaction (PCR) method. Ten virulence genes were studied: *kpsMT* K1*, ibeA, astA, iss, irp2, papC, iucD, tsh, vat,* and *cva/cvi*. The single PCR reactions for *kpsMT* K1 and *ibeA* were performed as described by Johnson et al. [14]. The presence of eight virulence factor genes - *astA, iss, irp2, papC, iucD, tsh, vat,* and *cva/cvi* was studied using the multiplex PCR method by Ewers et al. [15]. All amplifications were performed in a 25 μl reaction volume, and a lysate of overnight culture in sterile water was used as a template.

### Phylogenetic and O typing

*E. coli* isolates were assigned to phylogenetic groups using the PCR method documented earlier [16,17]. Based on the combination of genes (*svg*, *chuA*, *yjaA*, *uidA* and *TspE4.C2*) they possess, the isolates were assigned to one of five groups (A, B1, B2, D, B2_1_/ST29). *E. coli* O antigen groups O1, O2 and O18 were determined according to Clermont et al. [18]. Amplifications were performed in a 25 μl reaction volume, and a lysate of overnight culture in sterile water was used as a template.

### Control strains

The following *E. coli* strains were used as positive PCR controls: RS 228 and A56 (K1, O18 *ibeA)*; RS 228 (positive for all five genes), and ATCC 25922 (positive for all five genes except *svg)* for the phylogenetic groups*;* A110 (*astA*, *irp2, papC*); EC206 (*iss, irp2, papC*, *iucD, tsh, cva)*; 252 (*iss, irp2, papC, iucD, vat, cva,* O2); IH11002 (O1).

### Antimicrobial susceptibility testing

The susceptibility of 48 potential ExPEC isolates for nalidixic acid (30 μg) and ciprofloxacin (5 μg) was tested according to the standard Disk Susceptibility method [19]. The *E. coli* strains were also screened phenotypically for the production of ESBL with the double disk synergy test. Disks (Oxoid) containing cefotaxime, ceftazidime, and equivalent disks with clavulanic acid were used according to the standard method [20].

### Statistical analysis

Comparisons of proportions for virulence factors detected and the distribution of the strains to phylogenetic groups were analyzed by Chi-square test. Statistical significance was set at a P value of < 0.001.

## Results

*E. coli* was isolated from 207 (94.5%) of the 219 poultry meat products. One *E. coli* isolate from each product was characterized further. The majority of the isolates belonged to the phylogenetic group D (105; 50.7%), followed by groups A (78; 37.7%), and B2 (6; 7.7%) (Table 1). There were more phylogenetic group D and fewer group A isolates from marinated meat than from non-marinated meat, but the differences were not statistically significant. Two (1%) B2 isolates were positive for the *svg* gene specific for the clonal group B2_1_/ST95 (Table 1). They both belonged to O1 serogroup.

**Table 1:** **Phylogenetic distribution of the 207 *Escherichia coli *isolates originating from marinated and non-marinated retail poultry meat at Finnish retail market**

**Phylogenetic group**	**Non-marinated meat (*n*** **= 40)**	**Marinated meat (*n*** **= 167)**	**Total (*n*** **= 207)**
uidA negative ^ns^	0	1 (0.6%)	1 (0.5%)
A ^ns^	21 (52.5%)	57 (34.1%)	78 (37.7%)
B1 ^ns^	1 (2.5%)	4 (2.4%)	5 (2.4%)
D ^ns^	15 (37.5%)	90 (53.9%)	105 (50.7%)
B2 ^ns^	3 (7.5%)	13 (7.8%)	16 (7.7%)
B2_1_/ST29 ^ns^	0	2 (1.2%)	2 (1%)

^ns^ statistically non-significant.

In all isolates, we found at least one of the tested virulence genes (Table 2). Among the 123 strains of the phylogenetic groups B2 and D, the most common virulence genes were *iucD* (94 isolates*,* 76.4%), *irp2* (78 isolates, 63.4%), *iss* (40 isolates, 32.5%), and *astA* (75 isolates, 61%) (Table 2). The virulence factor genes were most frequent among isolates of phylogroup B2. Forty-eight (23.2%) of all isolates were positive for either two or all three of the genes classifying them as ExPEC: *iucD*, *papC* and *kpsMT* K1 (Table 3). ExPEC strains were as frequently detected from marinated (38/167; 22.8%) as from non-marinated (10/40; 25.0%) meat products.

**Table 2:** **Distribution of virulence factor genes (%) among 207 *Escherichia coli *–isolates belonging to major phylogenetic groups and isolated from poultry meat at Finnish retail market**

**Virulence factor genes**	**Prevalence of trait within phylogenetic groups, no. (%)**			**Total (*n*** **= 207)**
	**A + B1 (+uidA neg.) (*n*** **= 84)**	**D (*n*** **= 105)**	**B2 (+B2**_**1**_**/ST29) (*n*** **= 18)**	
cva*	26 (31%)	12 (11.4%)	10 (55.6%)	48 (23.2%)
vat*	1 (1.2%)	3 (2.9%)	10 (55.6%)	14 (6.8%)
tsh*	16 (19%)	2 (1.9%)	7 (38.9%)	25 (12.1%)
iucD*	29 (34.5%)	77 (73.3%)	17 (94.4%)	123 (59.4%)
irp2*	19 (22.6%)	62 (59%)	16 (88.9%)	97 (46.9%)
iss*	44 (52.4%)	28 (26.7%)	12 (66.7%)	84 (40.6%)
astA*	20 (23.8%)	73 (69.5%)	2 (11.1%)	95 (45.9%)
*papC*^*ns*^	7 (8.3%)	9 (8.6%)	5 (27.8%)	21 (10.1%)
ibeA*	0	2 (1.9%)	10 (55.6%)	12 (5.8%)
*kpsMT* K1*	0	40 (38.1%)	6 (33.3%)	46 (22.2%)

* *P* < 0,001; statistically significant.

^ns^ statistically non-significant.

**Table 3:** **Virulence gene profiles and phylogeny groups in ExPEC isolates (*n*** **= 48) and their distribution in marinated and non-marinated poultry meat at Finnish retail market**

**Combinations of genes**	**Phylogeny group**	**Non-marinated meat (*n*** **= 40)**	**Marinated meat (*n*** **= 167)**
*cva, vat, iucD, papC, irp2, iss, kpsMt* K1	B2*svg*^+^	0	2
*cva, vat, tsh, iucD, irp2, iss, ibeA, kpsMT* K1	B2	0	3
*cva, vat, tsh, iucD, papC, iss, astA*	D	0	1
*cva, iucD, irp2, iss, astA, kpsMt* K1	D	1	0
*cva, iucD, irp2, astA, kpsMt* K1	D	0	1
*vat, iucD, papC, irp2, iss, astA, kpsMT* K1	D	1	0
*iucD, papC, irp2, iss, astA, kpsMT* K1	D	0	6
*iucD, irp2, iss, astA, kpsMT* K1	D	2	4
*iucD, irp2, astA, kpsMT* K1	D	3	17
*iucD, iss, astA, kpsMT* K1	D	0	2
*iucC, papC, irp2, iss*	B2	2	0
*iucC, irp2*, *kpsMT* K1	D	1	0
*iucD, papC*	B2 and D	0	2
negative	various	30	129

The 48 isolates classified as ExPEC were susceptible to cephalosporins, ciprofloxacin and nalidixic acid, with the exception of one isolate (1/48; 2.1%) showing resistance to nalidixic acid. This ExPEC strain belonged to phylogenetic group D, and was positive for seven of the ten virulence factors studied.

## Discussion

In the present study, almost all Finnish retail poultry meat products were found to be contaminated with *E. coli*. Twenty-two per cent of the products contained *E. coli* strains that could be classified as ExPEC. The analysis of these poultry meat samples was focused to the virulence genes that are most typical of avian pathogenic ExPEC, and thus most likely to prevail in poultry products. Inclusion of all *kps*MT II group genes, instead of only that for K1 capsule, and some other ExPEC associated virulence factor genes, could have increased the observed prevalence of ExPEC. The findings from the Finnish poultry products are consistent with studies made in other countries on poultry meat at the retail level. Johnson et al. [2] found 21% of 110 *E. coli* isolates from chicken products to satisfy the criteria for ExPEC. Johnson et al. [6] reported a contamination rate of 46% for ExPEC in 189 retail poultry products. Xia et al. [21] found among different retail meats the highest rate of ExPEC strains in ground turkey (23.5%) and chicken breasts (20.2%). Kobayashi et al. [22] isolated 80 strains from 57 retail chicken carcasses, and grouped 21.25% of the strains into group B2 and 28.75% into group D. It has been recently shown that B2 strains positive for *svg* belong to a subset of ExPEC strains that contain avian pathogenic, uropathogenic as well as neonatal meningitis causing *E. coli*[23]. These strains often belong to serogroups O1, O2 or O18, and contain a high number of virulence genes. In this study, such ExPEC bacteria were found in 1% of Finnish poultry meat products. The isolates belonged to serogroup O1, and contained seven of the ten virulence genes studied.

Fecal contamination of carcasses at slaughter is presumed to be the source of contamination of meat with potential ExPEC bacteria [24-26]. However, our previous studies (unpublished) detected *E. coli* fulfilling ExPEC criteria only in 2.3% of fecal samples from healthy broilers. All these isolates belonged to phylogroup D, and none to B2. Our studies have shown that colibacillosis caused by avian pathogenic *E. coli* is not an uncommon disease in Finnish broiler houses. The study shows that such ExPEC bacteria can efficiently contaminate poultry meat and enter the food chain. In Finland, poultry meat is usually cut into pieces or very thin slices. It can be speculated that cutting may provide favorable or even selective conditions for the survival of ExPEC bacteria in the product consisting of meat and meat juice. The conditions may mimic those in the host tissue, thus supporting the survival of bacteria possessing protective virulence mechanisms. Meat processing may favor the survival of other types of *E. coli* as well*,* such ESBL-producing strains*,* in the meat products.

Marinades and especially their spices may potentially serve as sources of food pathogens, and furthermore promote pathogen survival. This has been a matter of concern in Finland, where most of the poultry meat is sold in marinades. In this study, we did not observe any differences between *E. coli* strains from marinated and non-marinated products with regard to prevalence of potential ExPEC and other virulence factor genes and phylogenetic groups. The studies on survival of *E. coli* in marinated meat products have focused on verocytotoxigenic strains. It has been stated that acidification of a traditional marinade resulted in greater lethality of *E. coli* O157:H7 compared with otherwise similar marinades that were not further acidified [27,28]. The pH of commercial marinades used in Finnish retail poultry meat products ranges from pH 4.16 ± 0.03 [29] to pH 4.5 [30]. It has been speculated that the buffering capacity of meat might neutralize the acidic components in marinades, leading to a decreased antimicrobial effect [8]. Although this study did not indicate that marinades are a risk factor for ExPEC contamination of the product, it is necessary to study the effect of marinades on survival of zoonotic *E. coli*, such as ESBL-producing strains.

In the present study, all potential ExPEC isolates (*n* = 48) were susceptible to ciprofloxacin and cephalosporins, and only one isolate exhibited resistance to nalidixic acid. These results illustrate the very low level of antimicrobials used in poultry production in Finland. Meat producing flocks, in particular, are usually reared without any antimicrobial treatment. The percentage of flocks treated has varied between 0.0 and 0.37% in broiler flocks and 3.79–4.97% in turkey flocks during 2007–2010 ( http://www.ett.fi). No fluoroquinolones or cephalosporins are used for poultry. Cephalosporin resistant *E. coli* isolates have emerged in numerous locations around the world after the sampling time of this study. This study, covering the Finnish retail market in 2006–2007, serves as a starting point for further studies. It is essential to gain more information about the antibiotic resistance and the genetic properties of the *E. coli* isolates present in retail poultry meat in Finland.

## Conclusions

Potential ExPEC bacteria were found in 22% of marinated and non-marinated poultry meat products on the Finnish retail market. Of the products, 0.9% were contaminated with *E. coli* [O1] B2 *svg*^+^ group, which has been implicated in multiple forms of ExPEC disease. There were no differences between marinated and non-marinated products with regard to the prevalence of potential ExPEC bacteria, other virulence factor genes and phylogenetic groups of the *E. coli* isolates. Poultry meat products on Finnish retail market may have zoonotic potential.

## Competing interests

The authors declared that they have no competing interests.

## Authors’ contribution

UL, PPM participated in the discussion on the study design. PPM, UL participated in the collection of samples, analysis and interpretation of the data. UL carried out the microbiological analyses of the samples. TP, KR and II carried out all used PCR methods and the antimicrobial susceptibility testing. Analysis and interpretation of the PCR and the antimicrobial susceptibility testing were carried out by TP, SP, II and KR. II and TP carried out the statistical analyses. UL, II, TP and SP wrote the manuscript. All authors read and approved the final manuscript.

## References

[B1] Russo TA Johnson JR Proposal for a new inclusive designation for extraintestinal pathogenic isolates of Escherichia coli: ExPEC J Infect Dis 2000 181 1753 1754 10.1086/315418 10823778

[B2] Johnson JR Murray AC Gajewski A Sullivan M Snippes P Kuskowski MA Smith KE Isolation and molecular characterization of nalidixic acid-resistant extraintestinal pathogenic Escherichia coli from retail chicken products Antimicrob Agents Chemother 2003 47 2161 2168 10.1128/AAC.47.7.2161-2168.2003 12821463 PMC161843

[B3] Manges AR Smith SP Lau BJ Nuval CJ Eisenberg JNS Dietrich PS Riley LW Retail meat consumption and the acquisition of antimicrobial resistant Escherichia coli causing urinary tract infections: a case–control study Foodborne Pathog Dis 2007 4 419 431 10.1089/fpd.2007.0026 18041952

[B4] Smith JL Fratamico PM Gunther NW Extraintestinal pathogenic Escherichia coli Foodborne Pathog Dis 2007 4 134 163 10.1089/fpd.2007.0087 17600482

[B5] Johnson JR Del Vari P O'Bryan TT Smith K Tatini S Contamination of retail foods, particularly turkey, from community markets (Minnesota, 1999–2000) with antimicrobial-resistant and extraintestinal pathogenic Escherichia coli Foodborne Pathog Dis 2005 2 38 49 10.1089/fpd.2005.2.38 15992297

[B6] Johnson JR Kuskowski MA Smith K O'Bryan TT Tatini S Antimicrobial-resistant and extraintestinal pathogenic Escherichia coli in retail foods J Infect Dis 2005 191 1040 1049 10.1086/428451 15747237

[B7] Johnson TJ Logue CM Wannemuehler YM Kariyawasam S Doetkott C Debroy C White DG Nolan LK Examination of the source and extended virulence genotypes of Escherichia coli contaminating retail poultry meat Foodborne Pathog Dis 2009 6 657 667 10.1089/fpd.2009.0266 19580453 PMC3145168

[B8] Björkroth J Microbiological ecology of marinated meat products Meat Sci 2005 70 477 480 10.1016/j.meatsci.2004.07.018 22063746

[B9] Taylor NM Davies RH Ridley A Clouting C Wales AD Clifton-Hadley FA A survey of fluoroquinolone resistance in Escherichia coli and thermophilic Campylobacter spp. on poultry and pig farms in Great Britain J Appl Microbiol 2008 105 1421 1431 10.1111/j.1365-2672.2008.03877.x 18778293

[B10] Costa D Vinue L Poeta P Coelho AC Matos M Saenz Y Somalo S Zarazaga M Rodrigues J Torres C Prevalence of extended-spectrum beta-lactamase-producing Escherichia coli isolates in faecal samples of broilers Vet Microbiol 2009 138 339 344 10.1016/j.vetmic.2009.03.029 19372011

[B11] Cortés P Blanc V Mora A Dahbi G Blanco JE Blanco M López C Andreu A Navarro F Alonso MP Bou G Blanco J Llagostera M Isolation and characterization of potentially pathogenic antimicrobial-resistant Escherichia coli strains from chicken and pig farms in Spain Appl Environ Microbiol 2010 76 2799 2805 10.1128/AEM.02421-09 20228098 PMC2863447

[B12] Randall LP Clouting C Horton RA Coldham NG Wu G Clifton-Hadley FA Davies RH Teale CJ Prevalence of Escherichia coli carrying extended-spectrum β-lactamases (CTX-M and TEM-52) from broiler chickens and turkeys in Great Britain between 2006 and 2009 J Antimicrob Chemother 2011 66 86 95 10.1093/jac/dkq396 21098542

[B13] Smet A Martel A Persoons D Dewulf J Heyndrickx M Catry B Herman L Haesebrouck F Butaye P Diversity of extended-spectrum beta-lactamases and class C beta-lactamases among cloacal Escherichia coli isolates in Belgian broiler farms Antimicrob Agents Chemother 2008 52 1238 1243 10.1128/AAC.01285-07 18227190 PMC2292556

[B14] Johnson JR Stell AL Extended virulence genotypes of Escherichia coli strains from patients with urosepsis in relation to phylogeny and host compromise J Infect Dis 2000 181 261 272 10.1086/315217 10608775

[B15] Ewers C Janssen T Kiessling S Philipp HC Wieler LH Rapid detection of virulence-associated genes in avian pathogenic Escherichia coli by multiplex polymerase chain reaction Avian Dis 2005 49 269 273 10.1637/7293-102604R 16094833

[B16] Clermont O Bonacorsi S Bingen E Rapid and simple determination of the Escherichia coli phylogenetic group Appl Environ Microbiol 2000 66 4555 4558 10.1128/AEM.66.10.4555-4558.2000 11010916 PMC92342

[B17] Bidet P Metais A Mahjoub-Messai F Durand L Dehem M Aujard Y Bingen E Nassif X Bonacorsi S Detection and identification by PCR of a highly virulent phylogenetic subgroup among extraintestinal pathogenic Escherichia coli B2 strains Appl Environ Microbiol 2007 73 2373 2377 10.1128/AEM.02341-06 17293507 PMC1855671

[B18] Clermont O Johnson JR Menard M Denamur E Determination of Escherichia coli O types by allele-specific polymerase reaction: application to the O types involved in human septicemia Diagn Microbiol Infect Dis 2007 57 129 136 10.1016/j.diagmicrobio.2006.08.007 17020797

[B19] Clinical and Laboratory standards institute Performance standards for antimicrobial disk and dilution susceptibility tests for bacteria isolated from animals; Approved standard – Third edition. CLSI document M31-A3. 28 2008 Wayne, PA, USA: Clinical and Laboratory standards institute Ref Type: Generic

[B20] Clinical and Laboratory standards institute Performance standards for antimicrobial susceptibility testing; sixteenth informational supplement. CLSI document M100-S16. 26 2006 Wayne, PA, USA: Clinical and Laboratory standards institute Ref Type: Generic

[B21] Xia X Meng J Zhao S Bodeis-Jones S Gaines SA Ayers SL McDermott PF Identification and antimicrobial resistance of extraintestinal pathogenic Escherichia coli from retail meats J Food Prot 2011 74 38 44 10.4315/0362-028X.JFP-10-251 21219761

[B22] Kobayashi RKT Aquino I Ferreira ALS Vidotto MC EcoR phylogenetic analysis and virulence genotyping of avian pathogenic Escherichia coli strains and Escherichia coli isolates from commercial chicken carcasses in Southern Brazil Foodborne Pathog Dis 2011 8 631 634 10.1089/fpd.2010.0726 21254888

[B23] Johnson TJ Wannemuehler Y Johnson SJ Stell AL Doetkott C Johnson JR Kim KS Spanjaard L Nolan LK Comparison of extraintestinal pathogenic Escherichia coli strains from human and avian sources reveals a mixed subset representing potential zoonotic pathogens Appl Environ Microbiol 2008 74 7043 7050 10.1128/AEM.01395-08 18820066 PMC2583479

[B24] Altekruse SF Elvinger F DebRoy C Pierson FW Eifert JD Sriranganathan N Pathogenic and Fecal Escherichia coli Strains from Turkeys in a Commercial Operation Avian Dis 2002 46 562 569 10.1637/0005-2086(2002)046[0562:PAFECS]2.0.CO;2 12243519

[B25] de Brito BG Carlos L Gaziri J Vidotto MC Virulence factors and clonal relationships among Escherichia coli strains isolated from broiler chickens with cellulitis Infect Immun 2003 71 4175 4177 10.1128/IAI.71.7.4175-4177.2003 12819112 PMC162012

[B26] Rodriguez-Siek KE Giddings CW Doetkott C Johnson TJ Nolan LK Characterizing the APEC pathotype Vet Res 2005 36 241 256 241 10.1051/vetres:2004057 15720976

[B27] Derrickson-Tharrington EL Evaluation of common acidulants for enhancement of destruction of Escherichia coli O157:H7 during drying of Gala apple slices and whole muscle beef jerky 2001 Fort Collins: Colorado State University MS thesis

[B28] Calicioglu M Sofos JN Samelis J Kendall PA Smith GC Inactivation of acid-adapted and non-adapted Escherichia coli O157:H7 during drying and storage of beef jerky treated with different marinades J Food Prot 2002 65 1394 1405 12233848 10.4315/0362-028x-65.9.1394

[B29] Isohanni P Alter T Saris P Lyhs U Wines as possible meat marinade ingredients possess antimicrobial potential against Campylobacter Poult Sci 2010 89 2704 2710 10.3382/ps.2009-00521 21076110

[B30] Perko-Mäkelä P Koljonen M Miettinen M Hänninen M-L Survival of Campylobacter jejuni in marinated and non-marinated chicken products J Food Saf 2000 20 209 216 10.1111/j.1745-4565.2000.tb00299.x

